# Crosstalk between the *Arabidopsis* Glutathione Peroxidase-Like 5 Isoenzyme (AtGPXL5) and Ethylene

**DOI:** 10.3390/ijms23105749

**Published:** 2022-05-20

**Authors:** Riyazuddin Riyazuddin, Krisztina Bela, Péter Poór, Ágnes Szepesi, Edit Horváth, Gábor Rigó, László Szabados, Attila Fehér, Jolán Csiszár

**Affiliations:** 1 Department of Plant Biology, Faculty of Science and Informatics, University of Szeged, Közép fasor 52., H-6726 Szeged, Hungary; riyazkhan24992@gmail.com (R.R.); bela.krisztina@szte.hu (K.B.); poorpeti@bio.u-szeged.hu (P.P.); szepesia@bio.u-szeged.hu (Á.S.); horvath.edit.03@szte.hu (E.H.); feher.attila@brc.hu (A.F.); 2 Biological Research Centre, Institute of Plant Biology, Temesvári krt. 62., H-6726 Szeged, Hungary; rigo.gabor@brc.hu (G.R.); szabados.laszlo@brc.hu (L.S.)

**Keywords:** antioxidant mechanism, *Arabidopsis thaliana*, ethylene, glutathione peroxidase-like 5 enzyme, reactive oxygen species

## Abstract

Glutathione peroxidases (GPXs) are important antioxidant enzymes in animals. Plants contain GPX-like (GPXL) enzymes, which—in contrast to GPXs—contain cysteine in their active site instead of selenocysteine. Although several studies proved their importance in development and stress responses, their interaction with ethylene (ET) signalling is not known. Our aim was to investigate the involvement of AtGPXL5 in ET biosynthesis and/or signalling using *Atgpxl5* mutant and *AtGPXL5* cDNA-overexpressing (OX-AtGPXL5) lines. Four-day-old dark-grown *Atgpxl5* seedlings had shorter hypocotyls and primary roots, while OX-AtGPXL5 seedlings exhibited a similar phenotype as wild type under normal conditions. Six-week-old OX-AtGPXL5 plants contained less H_2_O_2_ and malondialdehyde, but higher polyamine and similar ascorbate- and glutathione contents and redox potential (*E_GSH_*) than the Col-0. One-day treatment with the ET-precursor 1-aminocyclopropane-1-carboxylic acid (ACC) induced the activity of glutathione- and thioredoxin peroxidases and some other ROS-processing enzymes. In the *Atgpxl5* mutants, the *E_GSH_* became more oxidised; parallelly, it produced more ethylene after the ACC treatment than other genotypes. Although the enhanced ET evolution measured in the *Atgpxl5* mutant can be the result of the increased ROS level, the altered expression pattern of ET-related genes both in the *Atgpxl5* and OX-AtGPXL5 plants suggests the interplay between AtGPXL5 and ethylene signalling.

## 1. Introduction

The mammalian glutathione peroxidases (GPXs) are selenocysteine-containing non-heme thiol peroxidases that catalyse the reduction of H_2_O_2_ or organic hydroperoxides to water or corresponding alcohols using reduced glutathione (GSH) or thioredoxin (TRX) [1]. GPXs are central components of reactive oxygen species (ROS)-processing mechanisms and participate in the maintenance of membrane integrity [2,3]. The plant glutathione peroxidases are closely related to the animal GPX4 isoenzymes that are phospholipid hydroperoxide glutathione peroxidases [4]. However, the plant isoenzymes contain cysteine in their active site and most of them prefer TRX as an electron donor rather than GSH [5,6]. Because they more efficiently reduce peroxides different from H_2_O_2,_ such as lipid peroxides, the name GPX-like (GPXL) was introduced to designate the *Arabidopsis thaliana* GPX isoforms [7,8]. The *Arabidopsis* possesses eight GPXL isoenzymes with different subcellular localisations [7].

It was suggested that AtGPXLs may function both as ROS scavengers and as redox transducers and can be the link between the GSH and TRX redox systems [9]. GPXLs may interact with other proteins and hence they might be redox sensors and have even signalling functions [10,11]. The analysis of *GPXL* gene expression disclosed that their transcript levels can increase under various environmental stresses [6,12,13,14,15]. The overexpression of *GPXL*s was shown to enhance the tolerance against abiotic stresses, such as salt and drought, osmotic stress, heavy metal and oxidative stress in different plant species, while in some cases, it decreased the resistance against biotic stressors [15,16,17,18]. GPXLs were implicated in the regulation of plant growth and developmental processes, such as the establishment of root architecture [19,20], shoot development [19,20], regeneration [20,21], stomatal control [11,22], photosynthesis [23], leaf morphology [22] and hormone signalling [11,17,20]. Passaia and her co-workers demonstrated the importance of AtGPXLs in the hormone-mediated regulation of lateral root development, revealing interactions between AtGPXLs and auxin, abscisic acid and strigolactone signalling [20].

Ethylene (ET) is a multifunctional gaseous phytohormone that regulates several developmental and physiological processes, such as leaf epinasty, senescence, flowering, fruit ripening, the triple response of etiolated seedlings, the inhibition of root growth and the formation of adventitious roots and root hairs [24,25,26,27,28]. ET plays a significant role in controlling the defence against biotic and abiotic stresses. In agreement, increased ET evolution was reported under various stresses [29,30]. The ET biosynthesis pathway consists of two central steps: (1) S-adenosyl-L-methionine (SAM) is converted into 1-aminocyclopropane-1-carboxylic acid (ACC) by ACC synthase (ACS) and (2) ET is produced from ACC by the ACC oxidase (ACO) [26]. Besides being the precursor molecule of ET, increasing evidence confirms the potential function of ACC as an ET-independent signalling molecule [31,32,33].

ET and polyamine (PA) biosynthesis are coupled via the common SAM substrate, and therefore it is widely believed that there is a tight correlation between these pathways [34,35,36]. PAs are essential N-containing biomolecules playing roles in plant development and stress responses (reviewed in [37]). The most important PAs are diamine putrescine (Put), triamine spermidine (Spd) and tetramine spermine (Spm). The biosynthesis of higher-order PAs, Spd and Spm, requires aminopropyl groups donated from decarboxylated SAM by S-adenosyl-L-methionine decarboxylase (SAMDC). Despite the potential competition of ET and PAs for SAM, the exact role of ACC or ET remains to be elucidated in the regulation of PA metabolism and the involvement of GPXL isoforms in this crosstalk is known.

Once ET is synthesised, it can diffuse throughout the plant and binds to ET receptors to stimulate the ET response. Based on the analysis of etiolated mutant seedlings with the altered triple response, an ET-signal transduction pathway has been proposed in *Arabidopsis thaliana* that involves five ET receptors, including ETHYLENE TRIPLE RESPONSE 1 (ETR1), ETR2, ETHYLENE RESPONSE SENSOR 1, (ERS1), ERS2 and ETHYLENE INSENSITIVE 4 (EIN4), as well as other important positive-signalling components, such as EIN2 and EIN3, and the negative-signalling component CONSTITUTIVE TRIPLE RESPONSE 1 (CTR1) [38,39,40,41]. Light-grown *Arabidopsis* seedlings possessing mutation related to ET signalling exhibited altered root-branching patterns: while the *etr1-1* and *ein2-5* mutants had more lateral roots, the *ctr1-1* plantlet had fewer lateral roots than the Col-0 wild type [28]. A less branched root phenotype was detected in the ET-overproducing *eto-1* mutant or after applying ACC to wild-type plants [28].

There is ample evidence that suggests the direct and indirect roles of ET in regulating the ROS homeostasis in plants [42]. ET production can orchestrate, or temporarily repress, the expression of genes encoding the key catalytic subunits of enzymes generating superoxide anions (Rboh-NADPH oxidase D and F) and being responsible for an oxidative burst [43]. Both the ACS and ACO proteins can be the rate-limiting enzymes of ET biosynthesis under certain conditions. Their transcription is highly regulated by a wide variety of developmental, hormonal and environmental factors [26,44,45]. ACS and ACO show tissue-specific expression and localisation patterns [46,47,48,49,50] and are under tight post-transcriptional and post-translational regulatory control [51].

Khan et al. reported that the exogenous application of the ET source ethephon resulted in increased GSH content that indicates the involvement of ET in the regulation of the cellular GSH concentration [52]. Interestingly, endogenously elevated or exogenously applied GSH positively affects ET biosynthesis by modulating the transcriptional and post-transcriptional regulations of the ACS and ACO enzymes [49]. GSH depletion resulted in decreased lateral root density and root meristem malfunction [53]. Genome-wide transcript profiling analysis uncovered that numerous redox-related genes including GPXLs are implicated in the interaction between the redox and hormonal signalling [53]. The differences we have found earlier in the phenotype and salt stress response of the *Atgpxl5-1* mutant and *AtGPXL5*-overexpressing plants also indicated a complex interaction among the membrane-localised GPXL5, GSH redox potential and plant growth [7,18]. The growth of the *Atgpxl5* mutant but not the *AtGPXL5*-overexpressing (OX-AtGPXL5) plants was delayed in the standard growth condition [18].

In this paper, we investigated the effect of ET on the development of the *Atgpxl5* and OX-AtGPXL5 seedlings, treating them with the ET-precursor ACC. It was observed that a lower number of lateral roots developed on 2-week-old mutant plantlets as compared to their wild type and OX-AtGPXL5 counterparts. Furthermore, the dark-grown *Atgpxl5* mutants exhibited an altered hypocotyl hook development. Thus, we aimed to investigate the effect of AtGPXL5 on ET biosynthesis and/or signalling. The ET evolution and the expression of selected ET-related genes, several oxidative stress parameters and antioxidant mechanisms were analysed in 6-week-old hydroponically grown ACC-treated plants. Our results indicated that ACC differently changed the GSH level, the redox potential, the activities of glutathione- and thioredoxin peroxidases and that of some other ROS-processing enzymes in the *Atgpxl5* mutant and AtGPXL5-overexpressor lines than in the wild type, but several physiological traits, among them the skotomorphogenesis, ethylene evolution and the redox potential of OX-AtGPXL5, were similar to that of the wild-type seedlings.

## 2. Results

### 2.1. AtGPXL5 Regulates Hypocotyl Hook Development and Seedling Growth

The growth parameters of in vitro grown 2-week-old seedlings of Col-0 wild type, *Atgpxl5-1* mutant and OX-AtGPXL5-overexpressor *Arabidopsis* lines were compared. It was revealed that the light-grown seedlings possessed a similar phenotype except with shorter primary roots and a lower number of lateral roots of the *Atgpxl5-1* mutant than the wild type or the OX-AtGPXL5 plants (Table 1).

**Figure 1:**
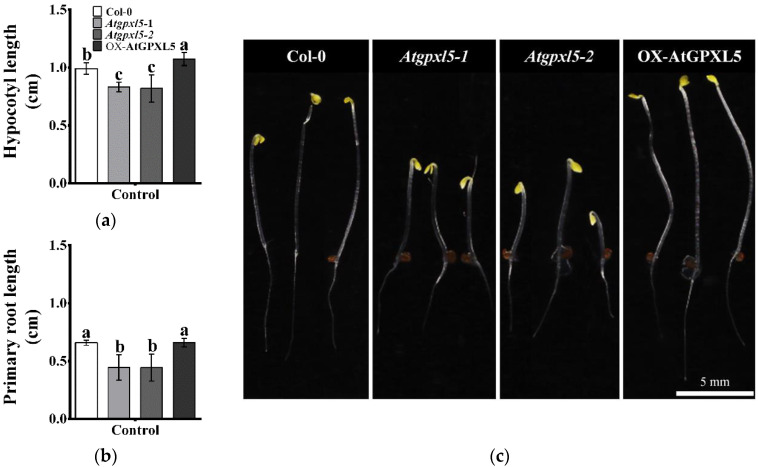
Skotomorphogenesis of *Arabidopsis* wild type (Col-0) and glutathione peroxidase-like 5 mutants (*Atgpxl5-1* and *Atgpxl5-2*) and overexpressing line (OX-AtGPXL5). The length of the hypocotyl (**a**) and primary root (**b**) of dark-grown seedlings and the overall phenotype of 4-day-old seedlings (**c**) germinated in dark. Mean ± SD, *n* = 15. Data were analysed using one-way ANOVA followed by Duncan’s test. Different letters represent data considered statistically significant at *p* ≤ 0.05.

The dark-grown *Atgpxl5* mutants were smaller; furthermore, they had an altered hypocotyl hook development compared to other genotypes (Figure 1). The different skotomorphogenesis of the *Atgpxl5* mutants and OX-AtGPXL5 seedlings raised the possibility of the crosstalk between the AtGPXL5 protein and the ET response.

### 2.2. The Level of the AtGPXL5 Affects the Ethylene Biosynthesis and Expression of Genes Involved in Ethylene Biosynthesis, Sensing and Signalling

The further evaluation of the relationship between AtGPXL5 and ET was performed on 6-week-old, hydroponically grown Col-0, *Atgpxl5-1* (*Atgpxl5*) and OX-AtGPXL5-1 (OX-AtGPXL5) plants. Under normal conditions, the ET production level was higher both in the shoots and roots of the *Atgpxl5* mutant compared to wild-type plants, while the ET evolution of the shoot of the overexpressing line was at the same level as the Col-0 (Table 2).

**Figure 2:**
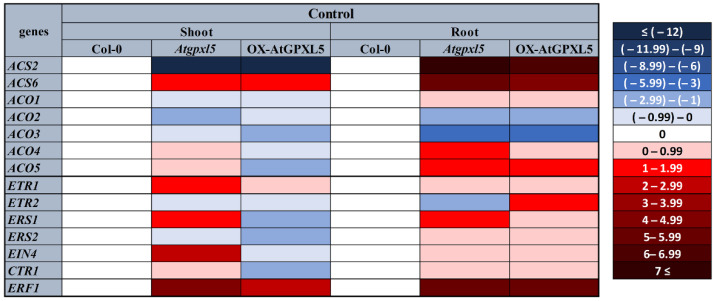
Relative expression of genes related to ET biosynthesis and signalling, such as *ACS2*, *ACS6*, *ACO1*, *ACO2*, *ACO3*, *ACO4*, *ACO5* and *ETR1*, *ETR2*, *ERS1*, *ERS2*, *EIN4*, *CTR1*, *ERF1*, in the shoots and roots of 6-week-old *Arabidopsis thaliana* wild type (Col-0), *Atgpxl5-1* insertional mutant (*Atgpxl5*) and AtGPXL5-overexpressor (OX-AtGPXL5) plants. The *actin2* (At3g18780) gene was used as internal control, data were normalised to the control wild-type values (1) and presented on a log2-scale as a heatmap. Red colours show activation, while blue colours show repression, according to the colour scale bar. The mean 2^(−ΔΔCt)^ data of RT-qPCR analysis are shown in Supplementary Table S1; *n* = 3. Abbreviations: ACO—ACC oxidase; ACS—ACC synthase; CTR—constitutive triple response; EIN—ethylene insensitive; ERS—ethylene response sensor; ETR—ethylene triple response.

The expression analysis of the genes implicated in the ET biosynthesis revealed AtGPXL5-dependent expression patterns and organ-dependent differences (Figure 2). In general, several investigated genes exhibited higher relative expressions in the roots than in the shoots of the *Atgpxl5* mutant as well as the OX-AtGPXL5 overexpressor in comparison to the expression of the respective organs in the Col-0 control. Two *ACS* genes, *ACS2* and *ACS6*, respectively, were selected for this investigation. Interestingly, both genes exhibited similar transcript-level changes in the mutant as well as the overexpressor in reference to the wild type. The *ACS2* gene exhibited a lower relative expression in the shoots but higher in the roots, while *ACS6* exhibited a higher relative expression in both organs of the two transgenic lines.

The *ACO* genes encode enzymes catalysing the final step of the ET biosynthesis. The expression analysis of five *ACO* genes showed the genotype and organ-dependent variability. For example, in the *Atgpxl5* mutant, the transcription of *ACO2* and *ACO3* were downregulated both in shoot and root compared to the Col-0; however, the transcript amounts of *ACO4* and *ACO5* were higher in the *Atgpxl5* mutant roots than in the Col-0 wild type (Figure 2). Interestingly, some *ACO* genes had similar expression-level changes in the mutant and the overexpressor in reference to the wild type (e.g., *ACO1* in both organs and *ACO2*, *ACO3* and *ACO5* in the roots), while the *ACO4* and *ACO5* relative expression was slightly increased in the mutant and decreased in the overexpressor shoot in reference to the wild type.

The expression of genes coding five ET receptors, such as *ETR1*, *ETR2*, *ERS1*, *ERS2* and *EIN4*, as well as other important signalling components, such as *CTR1* and *ERF1*, was also analysed. The *ETR1*, *ERS1*, *EIN4* and *ERF1* genes were highly upregulated in the shoot of the *Atgpxl5* mutant, while in the OX-AtGPXL5 shoots, some of them were even downregulated. In the roots, the transcript amount of *ERF1* was higher both in the *Atgpxl5* and OX-AtGPXL5 roots compared to the Col-0, while that of the *ETR2* changed differently in the two transgenic lines (Figure 2).

### 2.3. The Ethylene Precursor 1-Aminocyclopropane-1-Carboxylic Acid (ACC) Treatment Affects Differently the Growth of Investigated Plantlets

**Figure 3:**
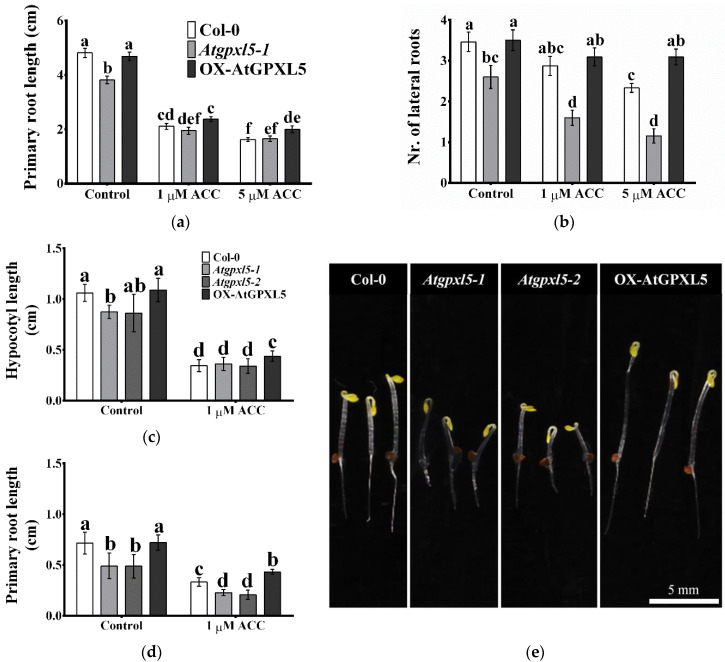
Growth of *Arabidopsis thaliana* wild type (Col-0) and glutathione peroxidase-like 5 mutants (*Atgpxl5-1* and *Atgpxl5-2*) and overexpressing line (OX-AtGPXL5) seedlings grown in light (**a**,**b**) or keeping them in dark (**c**–**e**) in the presence of the ethylene precursor 1-aminocyclopropane-1-carboxylic acid (ACC). The length of primary root (**a**) and the number of lateral roots (**b**) of two-week-old plantlets grown in vitro after transferring 5-day-old seedlings onto ½ MS media supplemented with one or five μM ACC. Length of the hypocotyl (**c**) and primary root (**d**) of dark-grown seedlings and the overall phenotype of 4-day-old seedlings germinated in dark in the presence of 1 μM ACC (**e**). Mean ± SD, *n* = 15. Data were analysed using one-way ANOVA followed by Duncan’s test. Different letters represent data considered statistically significant at *p* ≤ 0.05.

When seedlings were grown on culture media supplemented with 1 or 5 μM ACC for 10 days, the primary root lengths of all genotypes were reduced by ca. 50 to 70% (Figure 3a,b). The ACC treatments also decreased the number of lateral roots at all the investigated lines. The strongest reduction was detected on the *Atgpxl5* mutant (67% decrease with 5 μM ACC), while this treatment caused a rather small effect on the OX-AtGPXL5 plants (10% decrease) (Figure 3a,b).

The supplementation of the media with ACC triggered the triple-response phenotype (hampered hypocotyl and root elongation and the typically exaggerated hypocotyl hook phenotype) at the wild type and the OX-AtGPXL5 plantlets. However, the *Atgpxl5* mutant seedlings failed to display the exaggerated hook phenotype and had even smaller roots after treatment with 1 μM ACC. Although the OX-AtGPXL5 had a similar phenotype to the control, these plants had the highest hypocotyl and longest primary root and the least inhibition of the growth of the hypocotyls, both in the light and dark (Figure 3c–e, Supplementary Table S2). These results indicate crosstalk between the AtGPXL5 protein and the ET response.

### 2.4. The ACC Treatment Triggered the Highest ET Production in the Atgpxl5 Mutant

**Figure 4:**
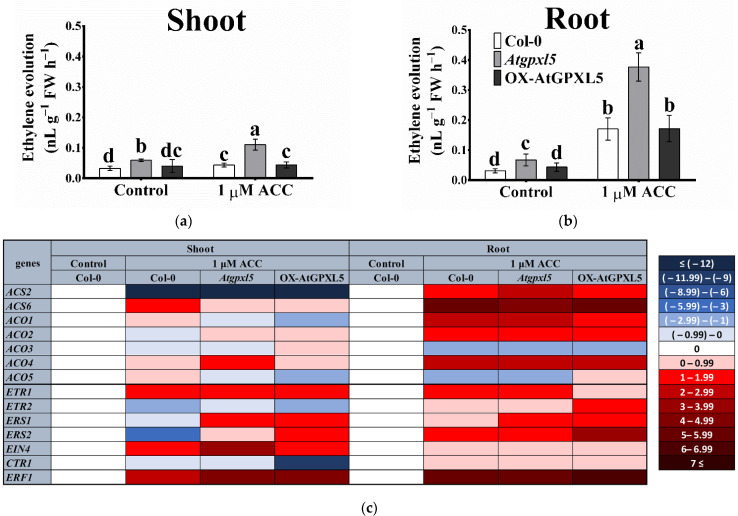
Effect of exogenous application of ACC on ET evolution (**a**,**b**) and relative expression of genes related to ET biosynthesis and signalling, such as *ACS2*, *ACS6*, *ACO1*, *ACO2*, *ACO3*, *ACO4*, *ACO5* and *ETR1*, *ETR2*, *ERS1*, *ERS2*, *EIN4*, *CTR1*, *ERF1* (**c**), in the shoots (**a**) and roots (**b**) of 6-week-old *Arabidopsis thaliana* wild type (Col-0), *Atgpxl5-1* insertional mutant (*Atgpxl5*) and AtGPXL5-overexpressing (OX-AtGPXL5) plants under control conditions and treated with 1 μM ACC for 24 h. The *actin2* (At3g18780) gene was used as internal control, data were normalised to the control wild-type values (1) and presented on a log2-scale as a heatmap. The mean 2^(−ΔΔCt)^ data of RT-qPCR analysis are shown in Supplementary Table S1; *n* = 3. Red colours show activation, while blue colours show repression, according to the colour scale bar. Abbreviations: ACC—1-aminocyclopropane-1-carboxylic acid; ACO—ACC oxidase; ACS—ACC synthase; CTR—constitutive triple response; EIN—ethylene insensitive; ERS—ethylene response sensor; ETR—ethylene triple response.

The exogenous application of 1 μM ACC to the Hoagland solution for 24 h elevated the ET evolution in the Col-0 and *Atgpxl5* shoots and in the roots of all the genotypes. The roots responded more strongly, likely due to their direct ACC uptake from the medium. The highest ET production was found in the *Atgpxl5* mutant shoots and roots (0.111 ± 0.018 and 0.377 ± 0.047 nL g^−1^ FW h^−1^, respectively). The ACC treatment induced the expression of *ACO4* in the *Atgpxl5* shoots, while in the roots, a rather variable expression pattern of the *ACS* and *ACO* genes was detected in the three genotypes. The *ACO1*, *ACO2* and *ACO4* genes were upregulated, while *ACS2* and *ACO5* were suppressed by the ACC treatment in the roots, which was similar in the genotypes tested (Figure 4). The ACC application reduced the expression of the *ACS* genes in all the genotypes, likely due to the feedback regulation.

Our results indicate that despite the similar regulation of the ET biosynthesis genes in the *Atgpxl5* mutant and OX-AtGPXL5 plants, the ET evolution is maintained on a similar level in the Col-0 and OX-AtGPXL5 plants even after the application of ACC, but it is elevated in the *Atgpxl5* mutants. Among the ET biosynthesis genes investigated, the relative expression of *ACO4* correlates best with an increased ET evolution in the mutant background, but the contribution of other mechanisms is indicated.

### 2.5. AtGPXL5 Isoenzyme Affects the Expression of Genes Involved in Ethylene Sensing and Signalling

The ACC treatment increased the *ETR1*, *EIN4* and *ERF1* transcription, but the expression of *ETR2* and *ERS2* was decreased in the shoot of wild-type Col-0 compared to the control conditions. In the shoot of the *Atgpxl5* mutant, the expression of all the investigated positive-signalling components was augmented after the ACC application. In the OX-AtGPXL5 shoots, the expression of the five genes (*ETR1*, *ERS1*, *ERS2*, *EIN4* and *ERF1*) was upregulated, while the transcription of *ETR2* and *CTR1* (the later one codes a negative regulator) was downregulated compared to the control conditions after 24 h. The application of 1 μM ACC evolved an even stronger effect on the selected genes in the roots of all the genotypes after 24 h. The highest impact of the ET precursor was detected in the OX-AtGPXL5 root, where the *ERS2* and *ERF1* (coding the transcription factor involved in ET response) transcription was induced most compared to the control conditions (Figure 4c).

### 2.6. The Ethylene/ACC Affects the GPOX, TPOX Activities

**Figure 5:**
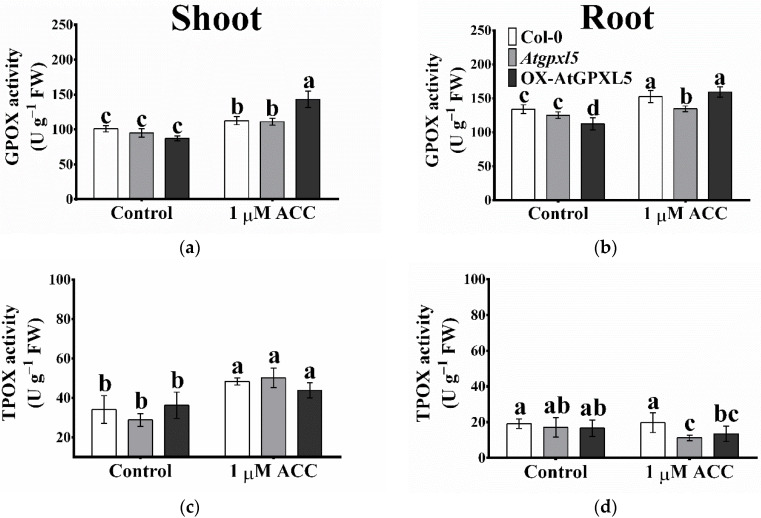
Effect of one-day 1 μM ACC treatment on the glutathione peroxidase (GPOX) activity (**a**,**b**) and thioredoxin peroxidase (TPOX) activity (**c**,**d**) in shoots and roots, respectively, of 6-week-old *Arabidopsis thaliana* wild type (Col-0), *Atgpxl5-1* insertional mutant (*Atgpxl5*) and AtGPXL5-overexpressing (OX-AtGPXL5) plants. Mean ± SD, *n* = 3. Data were analysed using one-way ANOVA followed by Duncan’s test. Different letters represent data considered statistically significant at *p* ≤ 0.05.

Under the control condition, there were no significant changes in the total extractable glutathione peroxidase (GPOX) activity of the *Atgpxl5* mutant shoot and roots, while it was slightly lower in the *AtGPXL5*-overexpressing roots than in the wild-type plants. The one-day treatment of ACC enhanced the GPOX activity of all (wild type, *Atgpxl5* mutant and OX-AtGPXL5) shoots and roots compared to the untreated conditions (Figure 5a,b). The thioredoxin peroxidase (TPOX) activity was not significantly different among the untreated plants. The precursor of ET elevated the TPOX activities in the shoot of all the genotypes compared to the control conditions. In the root, the TPOX activity remained unchanged or, in the case of the *Atgpxl5* mutant, even decreased compared to the untreated plants (Figure 5c,d).

### 2.7. ACC Treatment Reduced the H_2_O_2_ and MDA Accumulation in Atgpxl5 and OX-AtGPXL5 Plants

**Figure 6:**
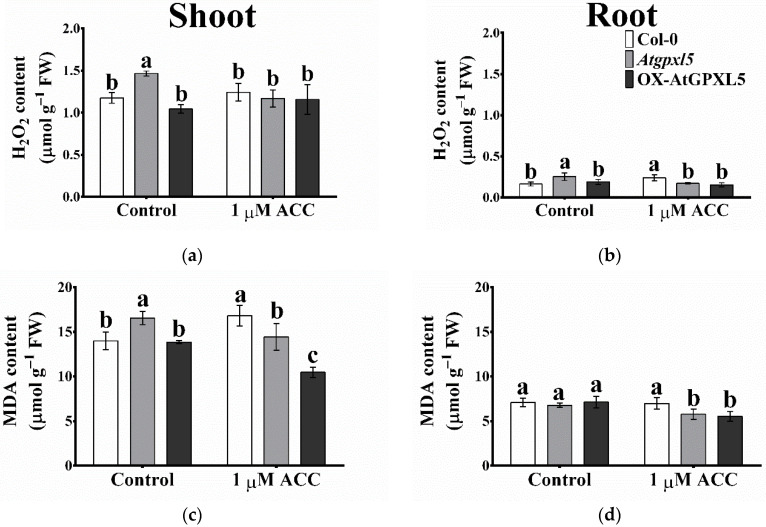
Effect of one-day 1 μM ACC treatment on the hydrogen peroxide (H_2_O_2_) (**a**,**b**) and malondialdehyde (MDA) contents (**c**,**d**) in shoots (**a**,**c**) and roots (**b**,**d**), respectively, of 6-week-old *Arabidopsis thaliana* wild type (Col-0), *Atgpxl5-1* insertional mutant (*Atgpxl5*) and AtGPXL5-overexpressing (OX-AtGPXL5) plants. The data are presented by mean values ± SD, *n* = 3. Data were analysed using one-way ANOVA followed by Duncan’s test. Different letters represent data considered statistically significant at *p* ≤ 0.05.

The analysis of the H_2_O_2_ level of the three genotypes under the control conditions showed significantly higher H_2_O_2_ content in the shoot and root of the *Atgpxl5* mutant compared to the other two lines. The ACC treatment elevated the H_2_O_2_ level only in the root of wild-type plants, while in the *Atgpxl5* mutant, it was even lower than under the control condition (Figure 6a,b). In the shoots, the amount of MDA in the *Atgpxl5* mutants was significantly higher than that in the Col-0 and OX-AtGPXL5 shoots. Moreover, the 1-day treatment of ACC elevated the MDA level in the Col-0 shoot, although in all transgenic shoots it was lower than under the control condition. In the roots, the application of ACC decreased the level of MDA in the *Atgpxl5* and OX-AtGPXL5 lines compared to the untreated plants (Figure 6c,d).

### 2.8. The Arabidopsis Glutathione Peroxidase-like 5 Interferes with Polyamine Metabolism

**Figure 7:**
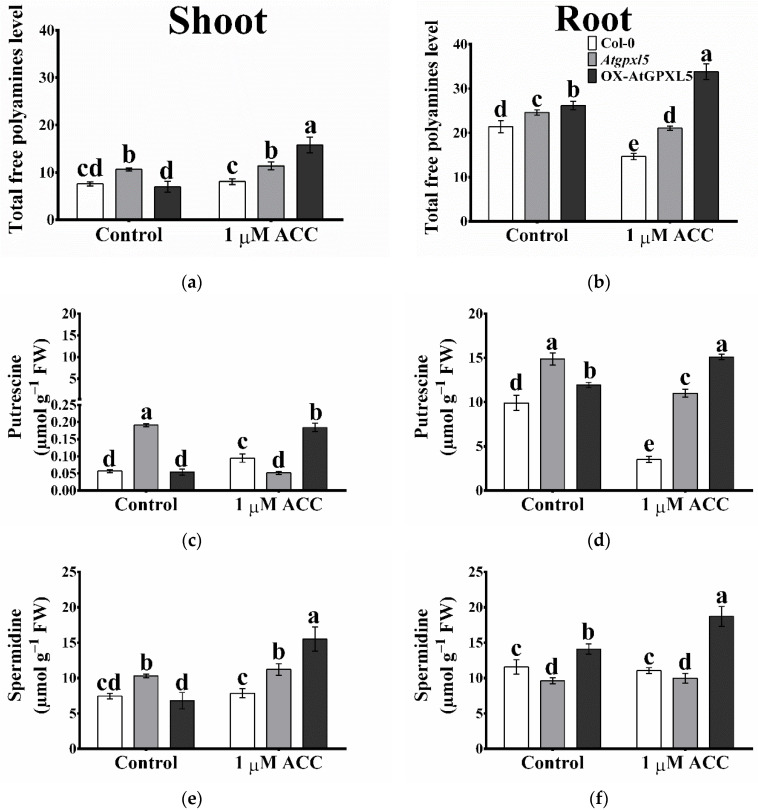

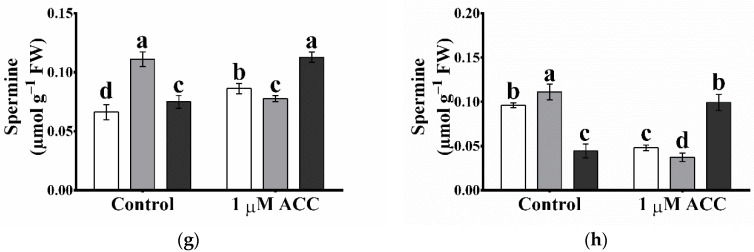
Effect of one-day 1 μM ACC treatment on free polyamine accumulation (**a**,**b**) (Put—putrescine (**c**,**d**), Spd—spermidine (**e**,**f**) and Spm—spermine (**g**,**h**)) in shoots (**a**,**c**,**e**,**g**) and roots (**b**,**d**,**f**,**h**) of Col-0, *Atgpxl5-1* insertional mutant (*Atgpxl5*) and AtGPXL5-overexpressing (OX-AtGPXL5) plants. The data are presented by mean values ± SD, *n* = 3. Data were analysed using one-way ANOVA followed by Duncan’s test. Different letters represent data considered statistically significant at *p* ≤ 0.05.

To investigate a possible connection between AtGPXL5, the ET production and PA metabolism, the contents of free PAs, Put, Spd, and Spm, were measured in 6-week-old Col-0, *Atgpxl5* and OX-AtGPXL5 plants under the control conditions as well as after the application of 1 μM ACC for 24 h. In normal conditions, both the root and the shoot of the *Atgpxl5* mutant showed higher total free PAs than the wild type. However, except for the slightly higher level of Spm, the *AtGPXL5*-overexpressing shoot had similar Put and Spd to the wild type under the control conditions (Figure 7). Measuring the activity of enzymes responsible for the PA catabolism (diamine oxidase and polyamine oxidase) did not show significant differences among the investigated genotypes indicating that the DAO and PAO enzymes are irresponsible for the increased H_2_O_2_ level in *Atgpxl5* mutants.

One-day-long 1 μM ACC treatment further elevated the level of Put and Spm and thus the total free PAs in the *AtGPXL5*-overexpressing shoots compared to the control conditions. Moreover, the exogenous application of ACC reduced the accumulation of Put and Spm in the *Atgpxl5* mutant shoots compared to the control conditions (Figure 7a,c,e,g). In untreated roots, the level of PAs in the *Atgpxl5* roots was higher despite the lower Spd content compared to the Col-0 and *AtGPXL5*-overexpressing plants. Interestingly, an even greater total free PA amount was measured in the *AtGPXL5*-overexpressing roots under the control conditions, regardless of the lowest Spm contents. The one-day treatment of ACC caused a reduction in the PA contents in the wild type as well as in the *Atgpxl5* mutant roots, except for the amount of Spd. Conversely, the ACC induced the highest accumulation of PAs in the overexpressing roots compared to the control condition (Figure 7).

### 2.9. Atgpxl5 Shoots Had Lower GSH Content and More Oxidised Glutathione Redox Potential Both under Control Conditions and after Application of ACC than Wild Type and OX-AtGPXL5 Plants

**Figure 8:**
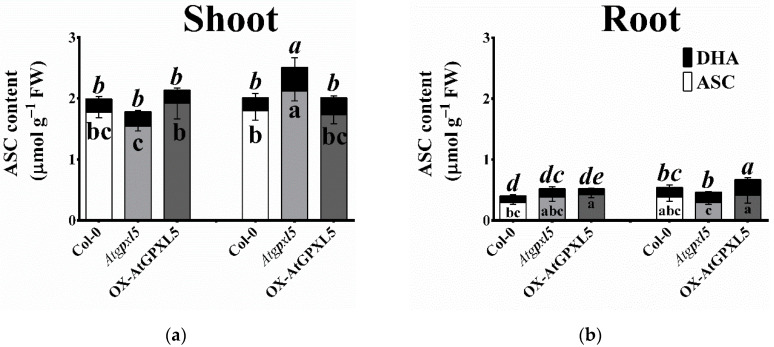

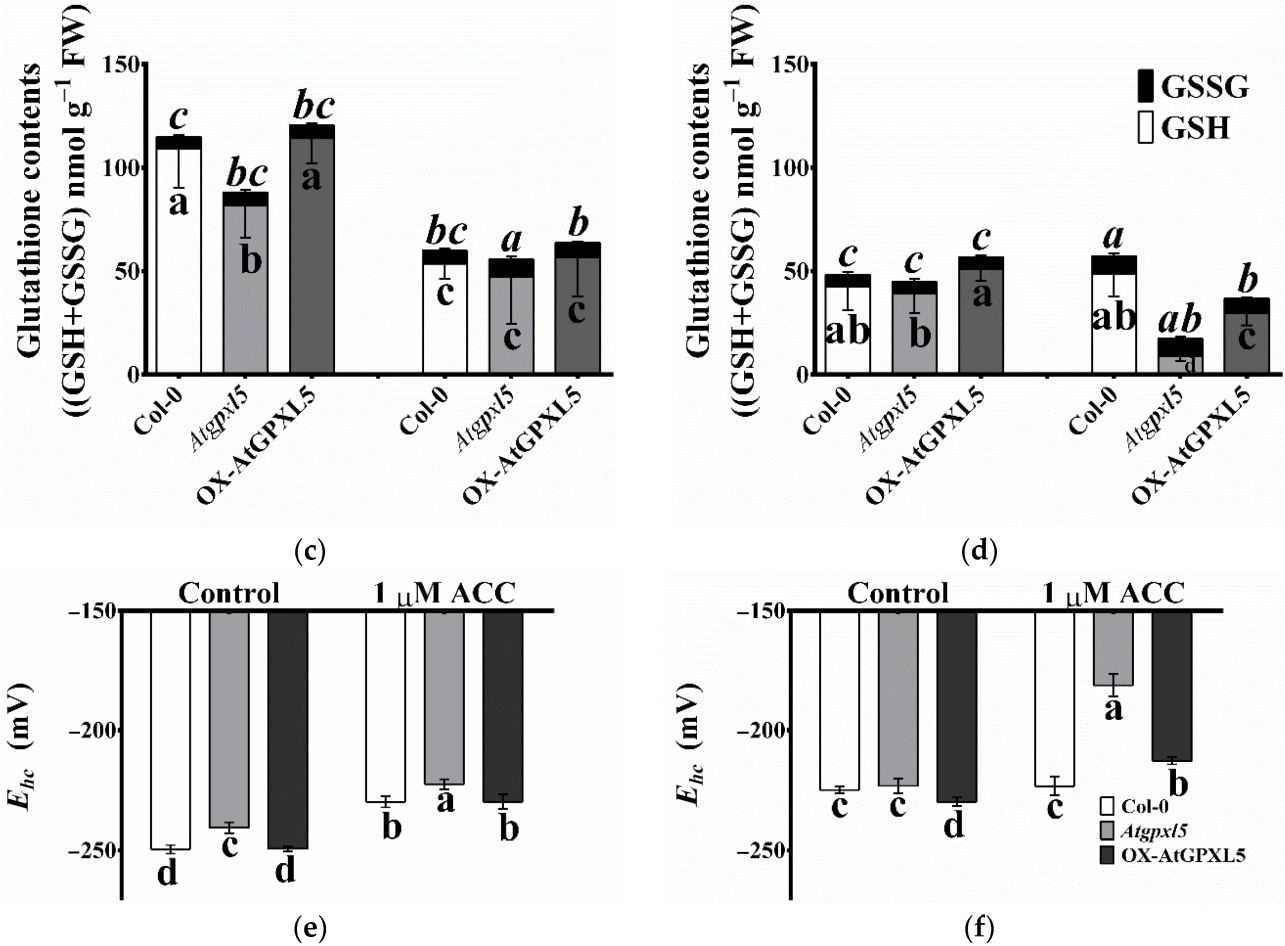
Effect of one-day 1 µM ACC on the ascorbate (**a**,**b**) and glutathione (**c**,**d**) contents, and the calculated glutathione half-cell redox potential (*E_hc_*; **e**,**f**) in shoots (**a**,**c**,**e**) and roots (**b**,**d**,**f**), respectively, of 6-week-old *Arabidopsis thaliana* wild type (Col-0), *Atgpxl5-1* mutant (*Atgpxl5*) and OX-AtGPXL5 plants. In the case of ASC and GSH contents, the dark segment of the bars represents the oxidised dehydroascorbate (DHA) and oxidised glutathione (GSSG), respectively. The data are presented by mean values ± SD, *n* = 3. Data were analysed using one-way ANOVA followed by Duncan’s test. Different letters represent data considered statistically significant at *p* ≤ 0.05. Normal lettering is used at the reduced and italics at the oxidised forms of antioxidants.

The comparison of the amounts of the main non-enzymatic antioxidants in 6-week-old hydroponically grown plants exhibited no clear tendency in the changes of the ASC or DHA levels among the investigated plants under the control conditions or after the ACC treatment. Significant differences were found between the higher ASC in the untreated OX-AtGPXL5 shoot compared to the *Atgpxl5* mutant shoot and between that of the OX-AtGPXL5 root compared to the wild type (Figure 8a,b). Applying 1 μM ACC increased the ASC and DHA level of the *Atgpxl5* shoot and the amount of DHA in the roots of all the genotypes compared to the untreated plants (Figure 8a,b).

Partly similar alterations were detected in the case of glutathione. Under the control conditions, the lowest GSH content was found in the *Atgpxl5* mutant, both in the shoots and roots. Except with the wild-type roots, the exogenous application of 1 μM ACC decreased the GSH level in the shoot and roots of plants. The lowest level of the GSH was measured in the *Atgpxl5* mutant roots, where the level of the GSH was reduced and became by ca. 72% lower than in the ACC-treated wild-type root (Figure 8c,d). The calculated *E_GSH_* reflected this pattern: while the most negative (reduced) values were detected in the untreated OX-AtGPXL5 shoot and root (−249.35 ± 0.93 and −229.89 ± 1.80 mV, respectively), the ACC-treated shoot and root of *Atgpxl5* plants became most oxidised (−222.49 ± 2.07 and −181.14 ± 4.7 mV, respectively) (Figure 8e,f).

### 2.10. The ROS-Processing Enzyme Activities Were Elevated Most Cases in Atgpxl5 Plants

**Figure 9:**
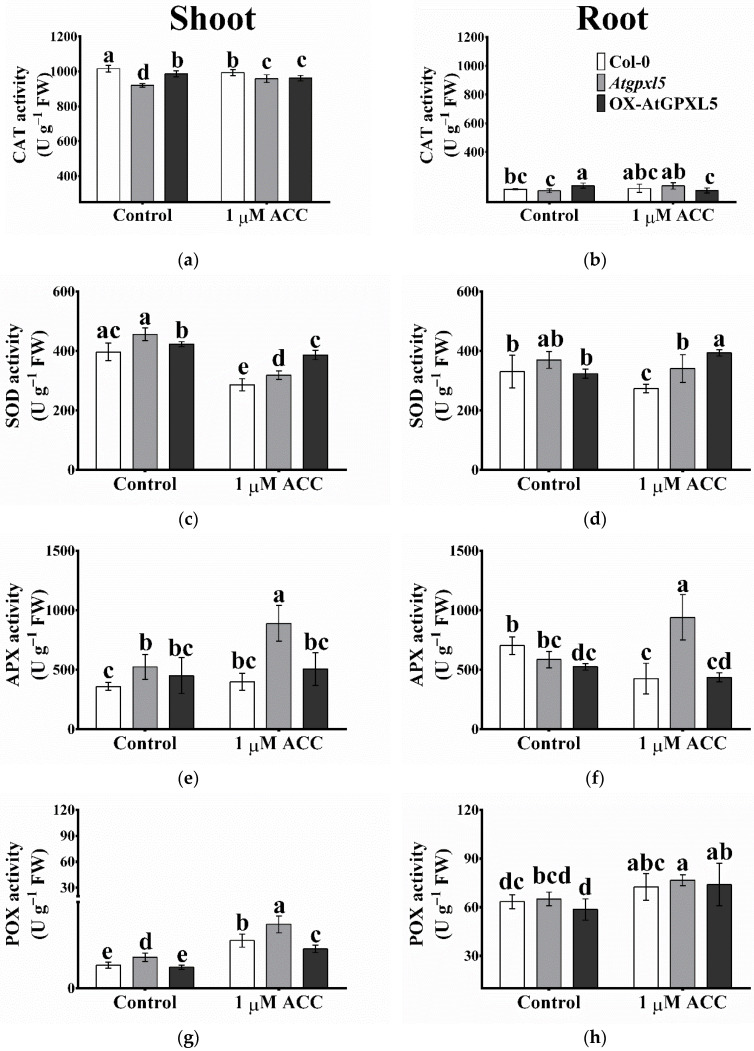

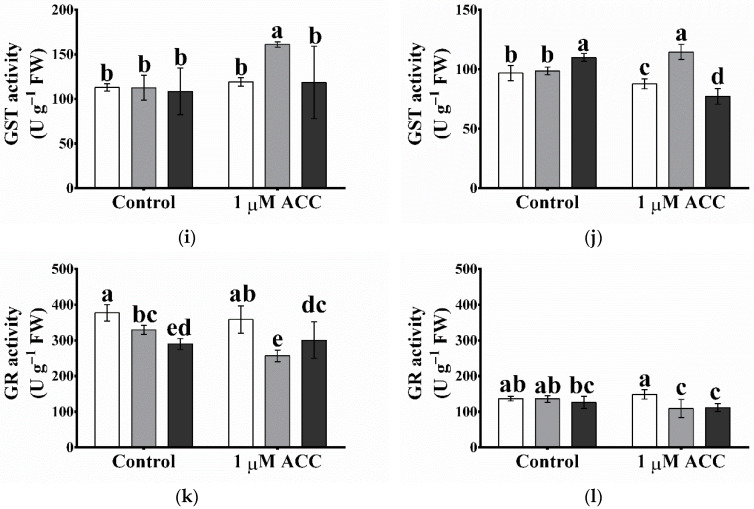
Effect of one-day 1 μM ACC treatment on the catalase (**a**,**b**), superoxide dismutase (**c**,**d**), ascorbate peroxidase (**e**,**f**), guaiacol peroxidase (**g**,**h**), glutathione transferase (**i**,**j**) and glutathione reductase (**k**,**l**) activities in shoots and roots, respectively, of 6-week-old *Arabidopsis thaliana* wild type (Col-0), *Atgpxl5-1* mutant (*Atgpxl5*) and OX-AtGPXL5 plants. The data are presented by mean values ± SD, *n* = 3. Data were analysed using one-way ANOVA followed by Duncan’s test. Different letters represent data considered statistically significant at *p* ≤ 0.05. n.s: statistically not significant.

Among the investigated H_2_O_2_-related enzyme activities, APX and POX were higher in the shoots of the *Atgpxl5* plants, while that of CAT and GR were lowered as compared to the Col-0 (Figure 9). In the overexpressing line, these peroxidases worked on the level of wild-type plants, the exception being the GR activity, which was lowered in the OX-AtGPXL5 shoots. The ACC treatment elevated the level of the CAT, APX, POX and GST activities and lowered the SOD and GR activities in the *Atgpxl5* shoots. In the case of overexpressing shoots, the ACC did not change these peroxidases, except for the higher activity of POX and the lower level of SOD activity. In the untreated roots, the OX-AtGPXL5 plants showed higher levels of CAT and GST and lowered APX activity than in the Col-0, but there were no changes in the antioxidant enzyme activities in the *Atgpxl5* roots. The application of ACC elevated the POX activity in the wild-type shoot and the SOD activity, especially in the OX-AtGPXL5 plants, but in the *Atgpxl5* plants, even the CAT, APX, POX, and GST activity was induced compared to the control conditions (Figure 9).

## 3. Discussion

### 3.1. The Relationship between AtGPXL5, Antioxidant Responses and ET

Ethylene is a well-known stress hormone that helps plants to survive under various biotic and abiotic stresses and is involved in multiple molecular and physiological plant processes, regulating various growth and cellular defence responses [54,55,56,57]. Interestingly, ET may exert its important role in abiotic stress tolerance via upregulation of *ACSs*, *ACOs* and other ET-signalling components [58,59,60,61]. The investigation of 6-week-old plants revealed that the *Atgpxl5* mutant produced more ET than wild-type and *AtGPXL5*-overexpressing plants; thus, the expression of genes involved in ethylene sensing and signalling was estimated.

It was found that the *Atgpxl5* shoots showed a higher level of ET and an increased transcript amount of genes encoding three ET receptors (*ETR1*, *ERS1* and *EIN4*). Although the post-transcriptional regulation of ET-signalling components has important significance [51], these results together with the mostly downregulated expression in the OX-AtGPXL5 line might indicate that *Atgpxl5* plants synthetise more receptors compared to the wild type and/or OX-AtGPXL5 plants under normal conditions. Interestingly, the expression of *ERF1* was outstandingly upregulated both in the *Atgpxl5* and OX-AtGPXL5 genotypes compared to the untreated wild type (Figure 2).

ERF transcription factors are involved in the activation of antioxidant defence and in the regulation of growth [57]. Both the increased ET level and oxidative stress induce *ERFs* and ET-induced ERFs are implicated in redox regulation [62]. Their crucial function was reported in salt, drought and heat stress tolerance by gene-specific regulations, where the ERF1 plays a nodal role in integrating the ET- and JA-signalling pathways [63]. Enhanced activities of antioxidant enzymes including SOD, CAT, APX, POX and reduced lipid peroxidation due to ACC treatment were reported in several plant species [64]. Similarly, the application of ethephon on mustard plants significantly increased the activity of antioxidant enzymes (SOD, APX, GR and GPX) along with reduced oxidative stress markers, such as H_2_O_2_ and MDA levels [42]. The increased GPOX and TPOX activities measured in our experiments after the ACC treatment indicate that AtGPXLs may be part of the ET signalling. Because AtGPXLs efficiently reduce lipid peroxides [5,6], it was suggested that the plasma-membrane-localised AtGPXLs (AtGPXL4 and AtGPXL5) have an important role in the maintenance of membrane integrity. Interestingly, the involvement of both ET and GPXL5 was proposed in the prevention in the recently described ferroptosis-like cell death in plants [65,66,67,68,69,70].

It was reported that *Arabidopsis* GPXL isoenzymes protect against abiotic stresses with the cooperation of enzymatic and non-enzymatic defence mechanisms [8,20]. Our earlier results indicated that the AtGPXL5 enzyme has a role in the fine-tuning of the GSH and ASC redox status and the level of ROS [8,18]. In the present experiments, the amount of H_2_O_2_ was also higher in the 6-week-old *Atgpxl5* mutant than in the wild type, but it also was demonstrated in the 4-day-old light-grown seedlings using a histochemical assay of DAB (1 3,3′-diaminobenzidine) to visualise the H_2_O_2_ content in the investigated genotypes (Supplementary Figure S1). Although the GPOX and TPOX activity did not decrease significantly, a lower GSH amount and CAT and GR activities were found in the mutant. At the same time, in the OX-AtGPXL5 plant, the GSH level was slightly elevated and the calculated *E_GSH_* values were about the level of the Col-0 or became even more negative under the control conditions (Figure 7). We have also found that GPXL5 affects PA biosynthesis, which also might result in the activation of antioxidant mechanisms [37], but the mechanisms resulting in PA accumulation either in the mutant and overexpressing plants needs further investigation. Based on the results of the PA metabolism investigations, it was concluded that the increase in the ROS level is not the consequence of their altered catabolism. Nevertheless, the enhanced ET evolution measured in the *Atgpxl5* mutant can be the result of the increased ROS level supporting the defence against oxidative stress, and the altered expression pattern of ET-related genes both in the mutant and *AtGPXL5*-overexpressing plants suggests that AtGPXL5 affects ET evolution and signalling. The similar redox status of the 6-week-old overexpressor and wild-type plants might explain that the OX-AtGPXL5 line does not have any phenotype and several of their physiological parameters are similar. The results of the experiments conducted on the OX-AtGPXL5 plants indicate a well-regulated transcription of the ET biosynthesis genes, though their control in the *Atgpxl5* plants might be compromised (Figure 2 and Figure 4).

### 3.2. The Interplay between ET, AtGPXL5, Redox Regulation and Growth Processes

The amount of GSH also affects the ET synthesis. By using plants with different endogenous GSH levels, Datta and her co-workers uncovered that overexpression of *AtECS1*, a GSH biosynthesis enzyme, resulted in a significant upregulation of genes involved in ET biosynthesis, including ACC synthase (*ACS2* and *ACS6*), and ACC oxidase (*ACO1*). In contrast, the downregulation of these genes was reported in a GSH-depleted *phytoalexin deficient2-1* (*pad2-1*) mutant [53]. *ACS2* and *ACS6* are often regulated by different stresses and can be the most abundant isoenzymes playing prominent roles in determining the ACS protein level [65,66]. Because the basal level of *ACO1* was higher in the *AtECS1*-overexpressing lines compared to the wild type, the increased content of ET observed in the transgenic lines suggested an intricate connection between ET and GSH. It was even hypothesised that GSH-mediated resistance to stresses occurs via an ethylene-signalling pathway [49].

On the other hand, ET also modulates the GSH synthesis and the redox state of plant cells. In *Brassica juncea*, ethephon reduced the oxidative stress and improved the photosynthetic performance in the presence of cadmium by decreasing stress-ethylene and elevating the GSH pool [52]. The involvement of ET in the regulation of GSH was reported under various abiotic stresses, such as heavy metal (Cd, Ni and Zn) [67,71], ozone [72] and salt stresses [25,73]. In the present study, except for wild-type roots, the ACC treatment decreased the amount of GSH and triggered more oxidised *E_GSH_*.

The low-molecular-weight GSH in a close partnership with ASC is a key player of the ROS homeostasis maintenance [74]. Because ROS are in tight interaction with the main plant hormones, they are thought to be core components of the complex regulatory network by integrating exogenous and endogenous signals, including hormones [75]. Redox processes, such as reduction–oxidation involving NADP-linked GSH and TRX systems, are involved in the regulation of interplay between auxin and strigolactone, and they affect auxin transport and signalling [76,77]. Passaia and her co-workers established the connection between the AtGPXLs and auxin, abscisic acid and strigolactone phytohormones by using *Atgpxl1*, *Atgpxl2*, *Atgpxl3*, *Atgpxl4*, *Atgpxl6*, *Atgpxl7* and *Atgpxl8* T-DNA insertion mutants, and they proved the importance of AtGPXLs in the hormone-mediated regulation of lateral root development [20]. According to our present results, the number of lateral roots of the 2-week-old light-grown *Atgpxl5* mutant seedlings was fewer compared to the other two genotypes. Moreover, the mutation affected the skotomorphogenesis of dark-grown seedlings, such as the length of the hypocotyl and radicle and the apical hook formation. The *Atgpxl5* mutants had reduced hypocotyl and radicle length compared to the wild type and OX-AtGPXL5, while the *AtGPXL5*-overexpressing seedlings had similar parameters than the wild type growing in the dark for 4 days (Figure 1). Moreover, the ACC treatment had the least effect on the dark-grown OX-AtGPXL5 seedlings. These data suggest that the AtGPXL5 protein has functions in the pathways that allow proper root and hypocotyl development (including apical hook formation), both in control conditions as well as after ACC treatment. Although the enhanced ET evolution measured in the 6-week-old *Atgpxl5* mutant can be the result of the increased ROS level, the altered expression pattern of ET-related genes both in the *Atgpxl5* and OX-AtGPXL5 plants suggests the interplay between AtGPXL5 and ethylene signalling.

Setting together these changes points to the central role of redox homeostasis and regulation. The shift in the redox state is a core component both in the activation of antioxidative mechanisms and the growth responses, e.g., through affecting PIN proteins [78] and transcriptional changes of key transcription factors, among them APETALA2 (AP2/ERF) and PLETHORA (PLT) [53]. Based on our results, we suppose that the changed redox potential, the increased GSH content and/or the elevated ET level are all involved in the different growth and stress responses of *Atgpxl5* and OX-AtGPXL5 plants.

## 4. Materials and Methods

### 4.1. Plant Material and Growth Conditions

*Arabidopsis thaliana* (L.) Heynh. ecotype Columbia (Col-0) as a wild-type control, T-DNA insertion mutants disrupting the *AtGPXL5* (*AT3G63080*) gene (*Atgpxl5-1*, SALK_076628C; *Atgpxl5-2*, *SAIL_720_A09*) and an *AtGPXL5*-overexpressing line (OX-AtGPXL5-1) were investigated [18]. Both the *Atgpxl5-1* and *Atgpxl5-2* harbour T-DNA insertion in their promoter (−286 and −888 positions from the start codon, respectively). Plants homozygous for the mutation were identified. The oligonucleotide sequences used in genetic analysis of the mutants were F: 5′ GAGATGAAGAAGGGCAAAGGT 3’, and R: 5′ TTGACGACGAGCAGCACT 3′. The additional used oligonucleotide in case of mutant obtained from Syngenta *Arabidopsis* Insertion Library was the SAIL-LB: 5′ AAATGGATAAATAGCCTTGCTTCC 3′. The relative expression levels of the *AtGPXL5* gene in the 6-week-old *Atgpxl5-1* and *Atgpxl5-2* mutants were 0.30 and 0.76 in their shoots, 0.36 and 0.41 in roots, respectively, compared to the wild type. *Arabidopsis* seeds were surface sterilised, stratified overnight at 4–8 °C and germinated on half-strength Murashige and Skoog medium (½ MS, Duchefa Biochemie; [79], with 0.5% sucrose and 0.8% agar) as was described in [8].

To test the effect of ET, three different experimental systems were employed. (i) “Light grown seedlings”. Five-day-old seedlings were transferred to square Petri dishes and plants were grown in vertically positioned plates in growth chamber (Fitoclima S 600 PLH, Aralab, Rio de Mouro, Portugal) at 21 °C, 100 μmol m^−2^ s^−1^ photon flux density, 10/14 h day/night period with 65% relative humidity. The length of primary root and number of lateral roots of 2-week-old seedlings were analysed with or without 1 or 5 µM ACC. Square Petri plates containing 5 plants from each genotype were scanned and root lengths were measured using ImageJ software (National Institutes of Health, Bethesda, MD, USA) [80]. Lateral roots were counted manually and the fresh weight (FW) of roots from each investigated line were determined [18]. Three plates per treatments were used in one experiment. (ii) “Dark-grown seedlings”. Triple response of 4-day-old seedlings of Col-0, *Atgpxl5* and OX-AtGPXL5 was investigated in vitro in the presence or absence of 1 μM ACC. After transferring seeds onto ½ MS media, the plates were kept in white light for 4 h to stimulate and synchronise seed germination [81], and seeds were germinated on vertical plates for 4 days. Seedlings were photographed and hypocotyl and primary root lengths were measured by the ImageJ software (National Institutes of Health, Bethesda, MD, USA) [80]. (iii) Hydroponic system. ET evolution and various physiological and molecular parameters were determined in 6-week-old plants cultured in a hydroponic system using Hoagland nutrient solution. Transcript levels of selected genes involved in ET biosynthesis and signalling, activities of the major enzymatic and non-enzymatic antioxidants and polyamine levels were measured. Samples were collected from fully expanded leaves and roots following the 1 μM ACC treatment for 24 h. The experiments were repeated at least two times, and the measurements were performed with three replicates unless indicated otherwise.

### 4.2. Ethylene Evolution Measurement

To determine the ET production, 500 mg freshly harvested shoot and root tissues were incubated in gas-tight glass flasks fitted with a rubber stopper. Five hundred μL distilled water was added to avoid tissue dehydration, and flasks were shaken in dark. A total of 2.5 mL of the gas volume was removed from the tubes with a gas-tight syringe after one hour and injected into a gas chromatograph ((Hewlett Packard 5890 Series II; Poway, CA, USA) equipped with a flame ionisation detector and activated alumina-filled column). Flow rates were 35 mL min^−1^ for He, 30 mL min^−1^ for H_2_ and 300 mL min^−1^ for air. The oven, injector and detector temperatures were 100, 120 and 160 °C, respectively [82]. ET standard was used for calculation of the amount of ET produced by the shoots and roots.

### 4.3. Determination of H_2_O_2_ Level and Malondialdehyde (MDA) Content

Measurements of H_2_O_2_ and the thiobarbituric acid (TBA)-reactive lipid peroxide amounts were performed as described in Bela et al. [8]. In case of H_2_O_2_ content determination, the absorbance of the samples was measured at 390 nm (Uvikon 930 spectrophotometer, Kontron AG, Eching, Germany), which was used for every absorption measurement in our experiments). The amount of H_2_O_2_ was calculated using a standard calibration curve prepared with a range of 0.1 to 5 mM H_2_O_2_ concentrations. To visualise the H_2_O_2_ content in the investigated genotypes, a histochemical assay was conducted on 4-day-old light-grown seedlings using 1 mg mL^−1^ 3,3′-diaminobenzidine (DAB) solution [83]. The MDA-level determination is based on the formation of TBA-MDA complex [84]. MDA concentrations were calculated using an extinction coefficient of 155 mM^−1^ cm^−1^.

### 4.4. Measurement of Free Polyamines

Polyamines were measured by high-pressure liquid chromatography (HPLC) as was published earlier in Szepesi et al. [85]. A total of 500 mg shoot or root tissues from 6-week-old plants was homogenised in pre-chilled mortar with 700 μL 5% perchloric acid (PCA), and the homogenate was incubated at 4 °C for 20 min. After centrifugation at 13,000× *g* for 20 min, 500 μL supernatant containing free polyamines was collected to a new glass test tube. A freshly prepared 400 μL 2 M NaOH and 10 μL benzoyl chloride was added to the supernatant. Following vortexing, the mixture was incubated at room temperature for 30 min. Benzoylated polyamines were extracted in 2 mL chilled diethyl ether and the clear upper organic phase was evaporated by a vacuum evaporator for total dryness. Samples in acetonitrile (ACN) were injected onto a reverse phase C18 column (250 × 4.6 mm internal diameter, 5 µm particle size; Phenomenex; Torrance, CA, USA). Analyses were performed by a JASCO HPLC system coupled with a UV–vis detector (JASCO HPLC System; Tokoyo, Japan) with a wavelength of 254 nm. The mobile phase consisted of water/ACN in 55:45 (*v*:*v*) ratio and the flow rate was 0.5 mL min^−1^. Peak area was used based on calculating concentrations of each polyamine standard purchased from Sigma-Aldrich, Darmstadt, Germany.

### 4.5. Enzymatic Antioxidant Activities

Glutathione peroxidase (GPOX; EC 1.11.1.9) and thioredoxin peroxidase (TPOX; EC 1.11.1.15) enzyme activities were measured spectrophotometrically with cumene hydroperoxide (CHP; Sigma-Aldrich, Darmstadt, Germany) substrate as was described by Riyazuddin et al. [18]. Compositions of GPOX reaction mixtures are the following: 4 mM GSH, 0.2 mM NADPH, 0.05 U of GR (from baker’s yeast, Sigma-Aldrich, Darmstadt, Germany), 100 μL enzyme extract and 0.5 mM substrate in a phosphate buffer (0.1 M, pH 7.0) in a total volume of 1 mL. In the case of TPOX, the reaction mixture contained 0.2 mM NADPH, 5 μM TRXh3, 0.1 μM NADPH-dependent thioredoxin reductase (NTRa) recombinant protein produced by *E. coli* according to Marty et al. [86], 50 μL of enzyme extract and 0.25 mM substrate in a Tris-HCl buffer (0.1 M, pH 7.4) in a total volume of 1 mL. One U was equal to nmol converted NADPH in 1 min, ε_340_ = 6.22 mM^−1^ cm^−1^.

### 4.6. Determination of ROS-Processing Enzyme Activities

To evaluate the activities of enzymes involved in ROS processing, 250 mg leaf and root tissues were homogenised on ice in 1 mL of 100 mM phosphate buffer (pH 7.0) containing 1 mM phenylmethylsulphonyl fluoride and 1% (*w*:*v*) polyvinyl-polypirrolidone, and the extracts were centrifuged at 13,000× *g* for 20 min at 4 °C.

Following the final step of extraction, the supernatant was used for measuring enzyme activities with the dual-beam spectrophotometer as was described by Riyazuddin et al. [18]. The superoxide dismutase (SOD; EC 1.15.1.1) activity was determined by measuring the ability of the enzyme to inhibit the photochemical reduction of nitro blue tetrazolium (NBT) in the existence of riboflavin in light [87]. One enzyme unit (U) of SOD was the amount that causes a 50% inhibition of NBT reduction in light. The catalase (CAT; EC 1.11.1.6) activity was determined by the decomposition of H_2_O_2_, measured spectrophotometrically by following the decrease in A_240_ [88]. The reaction mixture contained 100 μL tissue extract and 20 mM H_2_O_2_ in a phosphate buffer (50 mM, pH 7.0) in a total volume of 1.5 mL. One U is the amount of decomposed H_2_O_2_ (μmol) in 1 min, ε_240_ = 43.6 M^−1^ cm^−1^. The ascorbate peroxidase (APX; EC 1.11.1.11) activity was determined according to Tari et al. [89]. For the APX reaction, 1 mM ascorbate (Sigma-Aldrich) was added to the extraction buffer. The H_2_O_2_-dependent oxidation of ascorbate was followed by a decrease in A_290_. The reaction mixture contained 1 mM H_2_O_2_, 100 μL tissue extract and 50 μM ascorbate in a potassium phosphate buffer (50 mM, pH 7.0) in a total volume of 1 mL. One U was equal to nmol oxidised ascorbate in 1 min, ε_290_ = 2.8 mM^−1^ cm^−1^. The guaiacol peroxidase (POX; EC 1.11.1.7) activity was determined by monitoring the increase in A_470_ during the oxidation of the guaiacol substrate (Sigma-Aldrich, Darmstadt, Germany), according to Benyó et al. [90]. The 1.5 ml reaction mixture contained 30 mM H_2_O_2_ (Merck Millipore, Darmstadt, Germany), 10 μL tissue extract and 20 mM guaiacol substrate in a phosphate buffer (50 mM, pH 7.0). The amount of enzyme producing 1 μmol of oxidised guaiacol in 1 min was defined as 1 U, ε_470_ = 26.6 mM^−1^ cm^−1^. The glutathione transferase (GST; EC 2.5.1.18) activity was measured using the artificial 1-chloro-2,4-dinitrobenzene (CDNB; Sigma-Aldrich, Darmstadt, Germany) substrate [8]). The 1 mL reaction mixture contained 1 mM GSH, 100 μL tissue extract and 1 mM substrate in a phosphate buffer (0.1 mM, pH 6.5). One U was defined as the enzyme amount producing 1 nmol min^−1^ conjugated product in 1 min, ε_340_ = 9.6 mM^−1^ cm^−1^. GR (EC 1.8.1.7) activity was determined by measuring the changes in absorbance at 412 nm when 5,5′-dithiobis(2-nitrobenzoic acid) (DTNB) was reduced by GSH, generated from GSSG [91]. The activity was calculated as the amount of reduced DTNB in nmol min^−1^ g^−1^ FW, ε_420_ = 13.6 mM^−1^ cm^−1^.

### 4.7. Determination of Ascorbate and Glutathione Contents

Ascorbate (ASC) and glutathione (GSH) contents were measured as was described previously [18]. A total 300 mg of leaves or roots was homogenised on ice with 1.2 mL of 5% trichloroacetic acid (TCA). After centrifugation at 13,000× *g* for 20 min at 4 °C, the collected supernatant was used for further analysis. To measure the total level of ASC, 100 µL 10 mM dithiothreitol (DTT) was added to 100 μL supernatant. The excess DTT was eliminated by applying 100 µL 0.5% (*w*/*v*) N-ethylmaleimide (NEM). ASC concentrations were assessed in the mixture of 10% (*w*/*v*) TCA, 43% (*w*/*v*) H_3_PO_4_, 4% bipyridyl, 3% (*w*/*v*) FeCl_3_ spectrophotometrically at 525 nm. The quantity of the oxidised dehydroascorbic acid (DHA) form was determined by deducting the amount of reduced ascorbate from total ascorbate.

The total amount of GSH was detected using an enzymatic assay containing 100 mM phosphate buffer (pH 7.5), 1 mM DTNB, 1 mM NADPH, 1 U of GR (baker’s yeast, Sigma-Aldrich; Darmstadt, Germany) and 20 μL of the tissue extract from shoots and roots in a volume of 1 mL. GSH concentrations were measured spectrophotometrically at 412 nm. GSH content was estimated after the subtraction of oxidised glutathione from the amount of total glutathione.

The reduction potential of the GSH/GSSG couple (half-cell reduction potential; *E_hc_*) was calculated with the Nernst equation using the method of Schafer and Buettner [92]:

$$E_{hc}=-240-\left(59.1/2\right)\times \log({\left[\mathrm{GSH}\right]}^{2}/\left[\mathrm{GSSG}\right])\mathrm{mV}$$

where −240 mV is the standard reduction potential of glutathione on 25 °C, pH = 7.0.

### 4.8. RNA Extraction and Gene Expression Analyses

For the analysis of gene expression levels, 100 mg shoot or roots tissues from the frozen material were grounded in liquid nitrogen [8], and the RNA was isolated using the NucleoSpin RNA Plant and Fungi (Macherey, Dueren, Germany), following the kit guidelines. The genomic DNA was excluded by using RNA Clean & Concentrator (Zymo, Irvine, CA, USA) and the pure RNA free from DNA contamination was converted to cDNA by a reverse transcriptase (Thermo Fisher Scientific; Waltham, MA, USA) reaction. The cDNA was amplified using SYBR Green master mix (Thermo Fisher Scientific) with the Fast Real Time System (qTOWER Real-Time qPCR System, Analytik Jena, Jena, Germany) following the next protocol: denaturation at 95 °C for 7 min, followed by 40 cycles of denaturation at 95 °C for 15 s and annealing extension at 60 °C for 60 s. The primers used for the RT-qPCR are given in Supplementary Material (Supplementary Table S3). Data analysis was performed using qTOWER Software 2.2 (Analytik Jena, Jena, Germany) software and subsequent quantification was carried out by the CT method using *Actin2* (*At3g18780*) genes as internal controls. Data of RT-qPCR were calculated using the 2^(−ΔΔCt)^ formula [93]. Data were normalised to the control wild-type gene expression values (1) and presented on a log2-scale as a heatmap.

### 4.9. Statistical Analysis

Statistical analysis was carried out using SigmaPlot 11.0 software (SigmaPlot, Milano, Italy) by Duncan’s test and differences were considered at significant difference with 5% confidence (*p* ≤ 0.05). Means and standard deviation (±SD) were calculated from the data of at least 3 measurements, unless indicated otherwise. At least two biological and three independent technical replicates were performed in each experiment.

## 5. Conclusions

The AtGPXL5 protein is not only an antioxidant and redox fine-tuning enzyme, but it is involved in seedling development, including apical hook establishment under dark growth conditions. Interestingly, OX-AtGPXL5 seedlings at different ages exhibited under normal conditions a similar phenotype as the wild type. The physiological characterisation of 6-week-old lines revealed that OX-AtGPXL5 plants contained less H_2_O_2_ and MDA, but higher PA, similar ascorbate- and glutathione contents and redox potential (*E_GSH_*) than the wild type. In the *Atgpxl5* mutants, the *E_GSH_* became more oxidised; parallelly, it produced more ethylene than other genotypes. Although the enhanced ET evolution measured in the *Atgpxl5* mutant can be the result of the increased ROS level, the altered expression pattern of ET-related genes both in the *Atgpxl5* and OX-AtGPXL5 plants suggests the interplay between AtGPXL5 and ethylene signalling. The one-day treatment with the ET-precursor ACC induced the activity of the GPXL-related glutathione- and thioredoxin peroxidases and some other ROS-processing enzymes. The differences found in the etiolated seedlings and the ET response of the *Atgpxl5* mutant and *AtGPXL5*-overexpressing plants underline that AtGPXl5- and ET-related processes are interlinked.

## Figures and Tables

**Table 1:** The length of primary root and the number of lateral roots of two-week-old *Arabidopsis* wild type (Col-0) and glutathione peroxidase-like 5 mutant (*Atgpxl5-1*) and overexpressing line (OX-AtGPXL5) plantlets grown in light after transferring 5-day-old seedlings onto ½ MS media. Mean ± SD, *n* = 15. Data were analysed using one-way ANOVA followed by Duncan’s test. Different letters represent data considered statistically significant at *p* ≤ 0.05.

Growth Parameters	Col-0	*Atgpxl5*	OX-AtGPXL5
Root length (cm)	4.81 ± 0.17 a	3.81 ± 0.14 b	4.68 ± 0.15 a
Number of lateral roots	3.46 ± 0.24 a	2.60 ± 0.28 b	3.50 ± 0.26 a

**Table 2:** Ethylene evolution in the shoots and roots of hydroponically grown 6-week-old *Arabidopsis thaliana* wild type (Col-0), *Atgpxl5-1* insertional mutant (*Atgpxl5*) and AtGPXL5-overexpressor (OX-AtGPXL5) plants. Mean ± SD, *n* = 5. Data were analysed using one-way ANOVA followed by Duncan’s test. Different letters represent data considered statistically significant at *p* ≤ 0.05.

Ethylene Evolution (nL g^−1^FW h^−1^)	Col-0	*Atgpxl5*	OX-AtGPXL5
Shoot	0.033 ± 0.007 b	0.059 ± 0.004 a	0.040 ± 0.021 b
Root	0.031 ± 0.007 c	0.067 ± 0.020 a	0.043 ± 0.012 b

## Data Availability

Not applicable.

## Supplementary Materials

The following supporting information can be downloaded at: https://www.mdpi.com/article/10.3390/ijms23105749/s1.
