# Supplementary material for: Crosstalk between the Arabidopsis Glutathione Peroxidase-Like 5 Isoenzyme (AtGPXL5) and Ethylene
Source: Int J Mol Sci. 2022 May 20;23(10):5749. doi: 10.3390/ijms23105749 (PMC9171577; doi:10.3390/ijms23105749)
Supplement: Supplementary file 1 [file ijms-23-05749-s001.zip › ijms-1609900-supplementary.pdf]

Supplementary Material

Crosstalk between the *Arabidopsis* Glutathione Peroxidase-Like 5 Isoenzyme (AtGPXL5) and Ethylene

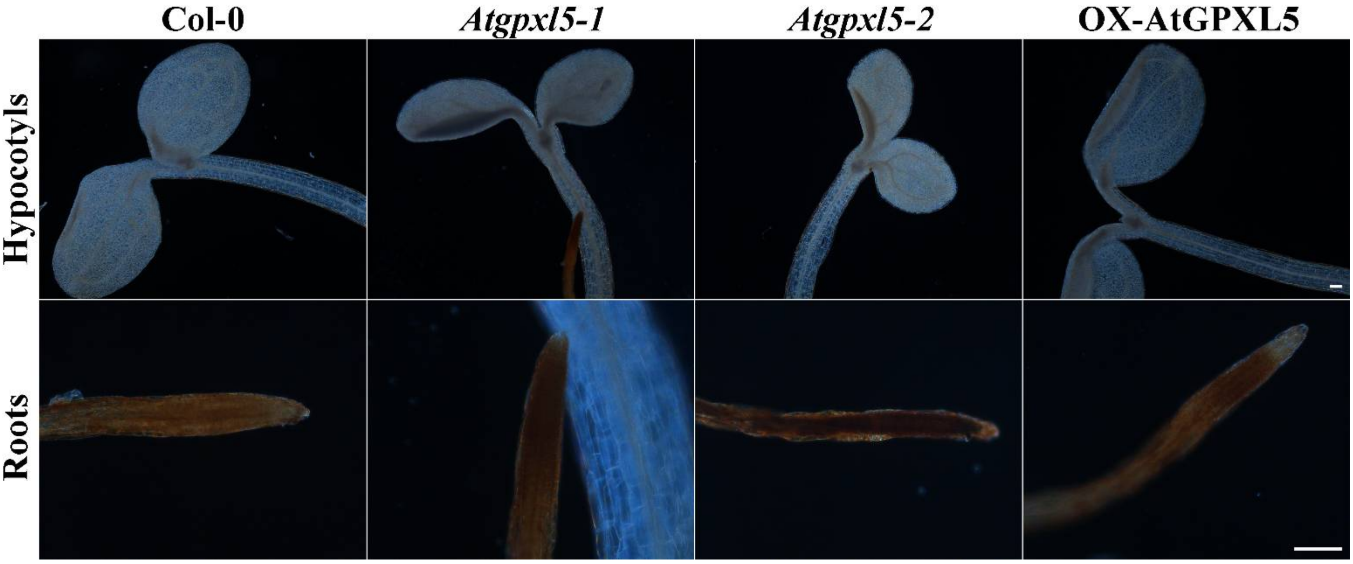

Figure S1. Histochemical assay of 4-day-old light-grown seedlings using DAB (3,3′-diaminobenzidine) staining to visualize the H_2O_2 content in the investigated genotypes (scalebar = 100 µm).

Table S1. The mean $2^{(-\Delta\Delta Ct)}$ data of RT-qPCR analysis.

Genes	Shoot						Root					
	Control			1 µM ACC			Control			1 µM ACC		
	Col-0	*Atgpxl5*	OX-AtGPXL5	Col-0	*Atgpxl5*	OX-AtGPXL5	Col-0	*Atgpxl5*	OX-AtGPXL5	Col-0	*Atgpxl5*	OX-AtGPXL5
ACS2	1	2.5×10^{-8}	6.7×10^{-10}	7.1×10^{-8}	1.8×10^{-8}	1.1×10^{-8}	1	332.57	69.31	2.61	4.26	2.17
ACS6	1	3.57	2.13	2.69	1.42	1.66	1	54.66	25.28	44.63	21.30	36.32
ACO1	1	0.70	0.58	1.53	0.84	0.18	1	1.17	1.67	5.54	6.83	2.16
ACO2	1	0.14	0.59	0.76	1.53	1.19	1	0.31	0.33	2.35	2.29	2.77
ACO3	1	0.90	0.34	0.97	0.69	1.40	1	0.12	0.08	0.19	0.20	0.36
ACO4	1	1.81	0.63	1.44	3.38	1.91	1	2.86	1.95	5.17	4.26	5.44
ACO5	1	1.22	0.40	1.06	0.68	0.44	1	2.54	2.68	0.24	0.27	1.75
ETR1	1	3.05	1.17	2.54	2.51	2.36	1	1.64	1.83	2.18	2.26	1.26
ETR2	1	0.55	0.89	0.24	0.93	0.49	1	0.36	2.81	1.20	1.99	2.34
ERS1	1	2.19	0.43	0.82	3.19	2.25	1	2.22	1.84	1.72	2.02	3.12
ERS2	1	0.96	0.26	0.11	1.49	2.70	1	1.94	1.40	2.85	3.45	8.53
EIN4	1	4.80	0.85	3.64	8.31	3.84	1	1.46	1.25	1.24	1.10	1.19
CTR1	1	1.05	0.24	0.87	0.80	5.5×10^{-4}	1	1.73	1.57	1.38	1.33	1.90
ERF1	1	17.60	4.05	5.47	24.34	20.04	1	37.60	43.56	32.22	54.10	78.11

Table S2. Responsiveness of the growth *Arabidopsis thaliana* wild type (Col-0) and glutathione peroxidase-like 5 mutants (*Atgpxl5-1* and *Atgpxl5-2*) and overexpressing line (OX-AtGPXL5) seedlings to 1 μM ACC (1-aminocyclopropane-1-carboxylic acid) treatment. The dark-gown seedlings were germinated and kept for 4 days in the presence of 1 μM ACC. The decrease of the hypocotyl and primary root length compared to untreated controls are calculated from data presented in Figure 3a.

	Control	1 uM ACC Treatment	Changes in Hypocotyl Length due to ACC Treatment (% of Control)	Changes in Primary Root Length due to ACC Treatment (% of Control)
	Ratio of Hypocotyl and Root Length	Ratio of Hypocotyl and Root Length		
Col-0	1.48 ± 0.20 ns	1.04 ± 0.13 b	32.46 ± 5.70 b	46.22 ± 5.77 b
Atgpxl5-1	1.78 ± 0.71 ns	1.62 ± 0.24 a	41.50 ± 7.38 a	45.71 ± 6.31 b
Atgpxl5-2	1.76 ± 0.67 ns	1.64 ± 0.30 a	39.57 ± 8.94 a	42.51 ± 7.01 b
OX-AtGPXL5	1.51 ± 0.15 ns	1.01 ± 0.06 b	40.20 ± 4.73 a	59.87 ± 3.37 a

Table S3. Primers pairs used for the quantitative real time PCR (RT-qPCR) analysis and the size of PCR products.

Genes	Forward Primers	Reverse Primers	Product Sizes	References
ACS2 (AT1G01480)	GGTGGTTATGAGCGGAGGA	TACGGGGAGGGAATGAGGA	93 bp	[1]
ACS6 (AT4G11280)	AGGCACGCTGAGATAACCAC	AATCCATCCACAAGAACAAACC	85 bp	[1]
ACO1 (AT2G19590)	TCAGATGCAGATTGGGAAAGC	TCTCACACATGAGCTTGGAGAGTC	160 bp	[2]
ACO2 (AT1G62380)	TCACCTCCCTCAATCCAATC	ATCCAACAAATCCTCAGCAAGA	112 bp	[1]
ACO3 (AT1G12010))	GACCCAGAAAGAAGGAAACAGG	AATGTCTCAACCACAGCCACC	240 bp	[3]
ACO4 (AT1G05010)	CTTGTGAAAACTGGGGCTTC	TGTTCCTTGGTCATCTTCTCC	88 bp	[1]
ACO5 (AT1G77330)	TGGATCGATGTTCAGCCTCTACC	TGCCACGCACTCTTGTACCTTC	95 bp	[4]
ETR1 (AT1G66340)	GTGTGTGTATGTGTGAGAGAGG	GCAATGAAGAAATCGGAGATG	131 bp	[1]
ETR2 (AT3G23150)	TCGTCCTCGCCTTCTACTTC	TCCCACATTTCTTCATCCAAG	149 bp	[1]
ERS1 (AT2G40940)	AACCACGAGATGAGGACACC	GCCACAAGATTGCTGCTTTT	125 bp	[1]
ERS2 (AT1G04310)	GTTCTGTGCTTGCCTCAGTG	ATGTGAATCGCCTCTGGTGT	115 bp	[1]
EIN4 (AT3G04580)	TGACTTTGATTCCGTTGCTG	TCATTTCCTTCTGCCTCTTCA	111 bp	[1]
CTR1 (AT5G03730)	CAGAAAATGGTGGTGGGTCT	TGTTTGGAACTGGCTGACTG	126 bp	[1]
ERF1 (AT3G23240.1)	TCCCTTCAACGAGAACGACTC	ACGGATTTGATCGGAAGGTC	111 bp	[2]
Actin2 (At3g18780)	GGTAACATTGTGCTCAGTGGTGG	AACGACCTTAATCTTCATGCTGC	108 bp	[5]
AtGPXL5 (At3G63080)	TCATCATCATCATCTGTGTCGGA	GGACTCCGTGAATCCGCATT	144 bp	[6]
GAPDH (At1G16300)	GAATCAACGGTTTCGGAAGA	CTCGGTGGTGATGAAAGGA	104 bp	[6]

References

1. Feigl, G.; Horváth, E.; Molnár, Á.; Oláh, D.; Poór, P.; Kolbert, Z. Ethylene-Nitric Oxide Interplay during Selenium-Induced Lateral Root Emergence in Arabidopsis. *J. Plant Growth Regul.* **2019**, *38*, 1481–1488.
2. Asensi-Fabado, M.A.; Cela, J.; Müller, M.; Arrom, L.; Chang, C.; Munné-Bosch, S. Enhanced Oxidative Stress in the Ethylene-Insensitive (Ein3-1) Mutant of Arabidopsis Thaliana Exposed to Salt Stress. *J. Plant Physiol.* **2012**, *169*, 360–368.
3. Lv, B.; Tian, H.; Zhang, F.; Liu, J.; Lu, S.; Bai, M.; Li, C.; Ding, Z. Brassinosteroids Regulate Root Growth by Controlling Reactive Oxygen Species Homeostasis and Dual Effect on Ethylene Synthesis in Arabidopsis. *PLoS Genet.* **2018**, *14*, e1007144.
4. Rauf, M.; Arif, M.; Fisahn, J.; Xue, G.-P.; Balazadeh, S.; Mueller-Roeber, B. NAC Transcription Factor Speedy Hyponastic Growth Regulates Flooding-Induced Leaf Movement in Arabidopsis. *Plant Cell* **2013**, *25*, 4941–4955.
5. Papdi, C.; Abrahám, E.; Joseph, M.P.; Popescu, C.; Koncz, C.; Szabados, L. Functional Identification of Arabidopsis Stress Regulatory Genes Using the Controlled CDNA Overexpression System. *Plant physiol.* **2008**, *147*, 528–542.
6. Riyazuddin, R.; Bela, K.; Horváth, E.; Rigó, G.; Gallé, Á.; Szabados, L.; Fehér, A.; Csiszár, J. Overexpression of the Arabidopsis Glutathione Peroxidase-like 5 Gene (AtGPXL5) Resulted in Altered Plant Development and Redox Status. *Environ. Exp. Bot.* **2019**, *167*, 103849.
